# Measuring the effect of COVID-19-related night curfews in a bundled intervention within Germany

**DOI:** 10.1038/s41598-022-24086-9

**Published:** 2022-11-17

**Authors:** Samuel de Haas, Georg Götz, Sven Heim

**Affiliations:** 1 Justus-Liebig-University Giessen, Giessen, Germany; 2 Mines ParisTech, Paris, France

**Keywords:** Viral infection, Health policy

## Abstract

We estimate the impact of local night curfews in Hesse, the fifth most populous federal state in Germany, on the growth of incidences of COVID-19 cases residing within the “second wave” of the pandemic. Thereby, we take advantage of the fact that all counties had the same measures in place with the only difference that some additionally had to implement night curfews due to state regulations. This allows us to identify the effect of night curfews as a salient part of a bundled intervention. In our case where different other measures are already in place, night curfews had at best a limited effect in slowing down the spread of the pandemic. The effect is not significantly different from zero.

## Introduction

Since the end of 2019 a new coronavirus SARS-CoV-2 spreads rapidly over the whole world and in early 2020 the WHO declared COVID-19 a pandemic^1^. After a slowdown in the summer of 2020 the “second wave” of the pandemic hit Europe, including Germany, very hard. In order to limit virus transmission, German authorities declared a lockdown from November 2, 2020. Parts of that lockdown were several non-pharmaceutical interventions (NPIs). Besides the implementation of nationwide measures, such as the limitation of gatherings and business closures, some regions with very high infection rates additionally imposed night curfews. While at that time there was a broad consensus on the effectiveness of NPIs in general and curfews in particular (e.g.^2,3^), the public debate about night curfews is highly controversial (e.g.^4^).

Similarly, there is also no consensus in the academic literature on whether night curfews present an appropriate measure to combat the pandemic. While some authors find that they are beneficial^5^, other studies are inconclusive or even find negative effects^6^. However, typically multiple NPIs are imposed simultaneously which makes it challenging to isolate the effect of a single intervention^7^ [Note that^5^ also report corresponding problems concerning the isolation of the effects of night curfews (p. 10): “However, due to the broad nature of these interventions, they are also likely to interact with other active NPIs.”].

In this study we examine the effectiveness of night curfews by taking advantage of regional and time variation in their implementation. Based on the federal system of Germany, NPIs were not imposed at the national level and even within federal states some NPIs were not imposed in all counties. In our analysis we use Hesse, the fifth most populous federal state in Germany, as a case study to assess the effectiveness of night curfews from 9pm to 5am which were only introduced in some but not all counties during the second wave. Using this setup we take advantage of the fact that counties in Hesse had the same measures in place, e.g., mask-wearing policies, restrictions of social contacts, restaurant and retail store closure. The only difference was that some additionally had to implement night curfews as part of a bundled intervention, which included some minor NPIs, which were imposed simultaneously with a night curfew. The most important ones are an alcohol ban in certain major public urban areas and the prohibition of indoor individual sports (e.g. indoor tennis). In terms of effectiveness of these two measures, one has to take into account that the former banned outdoor alcohol consumption in winter times at a few well-defined spots, but not in others. As far as the later intervention is concerned, tennis playing in indoor courts hardly affects many people in Germany. Given the limited nature of these interventions, we didn’t further inquire into their possible isolated effect. Even though we are therefore not able to disentangle the effect of these different measures from the effect of the night curfew, it is obvious that the night curfew is the salient part of this bundled intervention. As we find that the effect of the joint measures is at best limited and not statistically different from zero, we conclude that this also holds for the effect of the night curfew in isolation. Of course, this conclusion is based on the reasonable assumption that the other measures do not increase the incidences. Finally, note that our identification strategy makes use of the fact that night curfews were implemented at different points in time and with different durations. This peculiarity allows us to identify a potential effect by using a control group when measuring the treatment effects.

## Data and methodology

**Figure 1:**
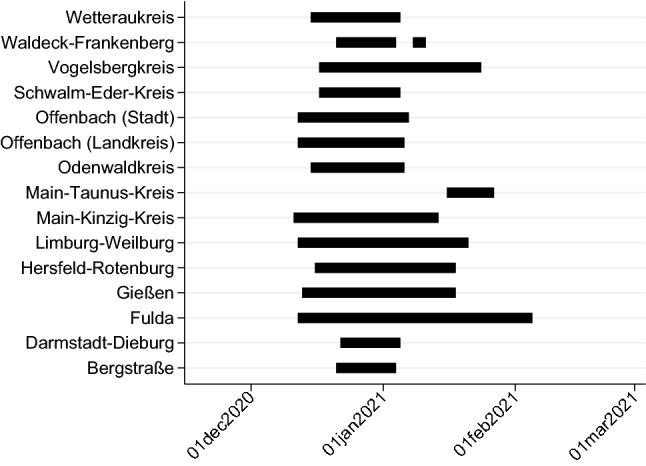
Night curfews in Hesse.

Our data set is built from two sources. Daily information on incidences (cumulative number of newly transmitted cases per 100,000 inhabitants over the past 7 days) at the county level were downloaded from the website of the Robert Koch Institute (RKI)^8^. Hessischer Rundfunk, the regional public broadcasting agency collected information on local night curfews in Hesse consisting of start and end dates per county. Our period of investigation starts on November 18, 2020 (when the RKI data start) and ends on February 28, 2021. This period resides within the second wave in Hesse. There are 26 counties of which 15 had a night curfew during our observation period. The average duration of a night curfew was 28 days. Figure 1 and Table 1 illustrates the timing of each night curfew and shows whether or not a curfew has been implemented.

**Table 1:** Night curfews in Hesse.

County	Start date	End date	Duration in days
Bergstraße	21/12/2020	04/01/2021	14
Darmstadt	–	–	–
Darmstadt-Dieburg	22/12/2020	05/01/2021	14
Frankfurt am Main	–	–	–
Fulda	12/12/2020	05/02/2020	55
Gießen	13/12/2020	18/01/2021	36
Groß-Gerau	–	–	–
Hersfeld-Rotenburg	16/12/2020	18/01/2021	33
Hochtaunuskreis	–	–	–
Kassel (Landkreis)	–	–	–
Kassel (Stadt)	–	–	–
Lahn-Dill-Kreis	–	–	–
Limburg-Weilburg	12/12/2020	21/01/2021	40
Main-Kinzig-Kreis	11/12/2020	14/01/2021	34
Main-Taunus-Kreis	16/01/2021	27/01/2021	11
Marburg-Biedenkopf	–	–	–
Odenwaldkreis	15/12/2020	06/01/2021	22
Offenbach (Landkreis)	12/12/2020	06/01/2021	25
Offenbach (Stadt)	12/12/2020	07/01/2021	26
Rheingau-Taunus-Kreis	–	–	–
Schwalm-Eder-Kreis	17/12/2020	05/01/2021	19
Vogelbergkreis	17/12/2020	24/01/2021	38
Waldeck-Frankenberg	21/12/2020	04/01/2021	14
Waldeck-Frankenberg	08/01/2021	11/01/2021	3
Werra-Meißner-Kreis	–	–	–
Wetteraukreis	15/12/2020	05/01/2021	21
Wiesbaden	–	–	–

The counties that had nighttime curfews and those that did not are similar in terms of some major characteristics such as population, population density, unemployment rate, real disposable income per capita, male-to-female ratio and average age. This is tested in two one sided *t*-tests of these characteristics, as shown in Table 2. The test statistics suggests that the counties that implemented nighttime curfews during our observation period and those that did not do not differ statistically in this regard.

**Table 2:** Two one sided *t*-test of counties with and without nighttime curfews.

	Mean	S.E.	*t*-Test I	*t*-Test II
			Curfew > no curfew	Curfew < no curfew
			(*p* Value)	(*p* Value)
**Population**				
Curfew	227,872	26,441		
No curfew	267,406	52,113		
			0.76	0.24
**Population density**				
Curfew	539.21	199.65		
No curfew	880.91	188.32		
			0.84	0.16
**Unemployment rate**				
Curfew	4.98	0.43		
No curfew	5.75	0.40		
			0.89	0.11
**Real disposable income per capita**				
Curfew	23,369	610		
No curfew	24,052	1227		
			0.70	0.30
**Average age**				
Curfew	44.6	0.42		
No curfew	43.9	0.66		
			0.17	0.83
**Male-to-female ratio**				
Curfew	0.98	0.004		
No curfew	0.97	0.009		
			0.13	0.87

*t*-test I gives the results of a *t*-test testing $H_{0}$ “the difference in means between counties that implemented a curfew versus counties that did not is equal to 0” versus $H_1$ “the difference in means between counties that did not vs counties that did implement a curfew not is larger than 0”. *t*-Test II tests the opposite alternative hypothesis. The county of Waldeck-Frankenberg is excluded for reasons discussed later. Data downdoaded from^9^.

To examine whether night curfews were effective in slowing down local incidence growth we apply a *difference-in-differences* approach. The idea is to asses whether incidence growth was smaller following a night curfew than it would have been in absence of it, by comparing the development of incidence growth in counties that have implemented night curfews with those that did not. A similar approach was used by^10^ and^11^ to examine the effects of several NPIs during the “first wave” in Germany.

As with all NPIs aiming to reduce incidences there is a notable time delay until a measure’s success can be evaluated. This is due to incubation period and delays in the recording and reporting of the incidence rates at the RKI website. The incubation period is assumed to be five days on average and the reporting lag adds two to nine days on top of that^12^. To account for the delay until night curfews actually unfold a measurable effect we move the start and end dates of each night curfew seven, fourteen and twenty-one days ahead of their real dates and construct a binary variable “Effective curfew” which is equal to one during this period and zero otherwise. In formal terms:

1 $$\begin{aligned} \begin{aligned} \text {Effective curfew}_{i,t}= {\left\{ \begin{array}{ll} 1, & \text { if }t\in [\text {Actual curfew start date}_{i} + \text {7/14/21 days};\\ & \text {Actual curfew end date}_{i} + \text {7/14/21 days}]\\ 0, & \text {otherwise} \end{array}\right. } \end{aligned} \end{aligned}$$

where *i* denotes counties and *t* days.

A major challenge in the identification of the effectiveness of night curfews comes from the fact that they have not been introduced randomly. On the contrary, night curfews have usually had to be implemented in counties in which the incidence exceeded a threshold of 200 on at least three consecutive days. [https://web.archive.org/web/20210130130947/https://www.hessen.de/fuer-buerger/corona-hessen/das-hessische-eskalationskonzept-im-ampelsystem] In other words, action was taken in counties with already higher incidences. While different incidence levels pre-curfew do not constitute a problem to identification, it may also be that incidence growth in counties that implemented night curfews already differed pre-curfew from those that did not implement a curfew. If the latter is the case the *common trend assumption*, i.e. homogeneity of incidence growth pre-curfew, would be violated and difference-in-differences estimation would fail to provide valid estimates of the the effectiveness of curfews.

To test the plausibility of the common trend assumption, we additionally include a binary variable into the model that indicates whether there were potential differences in pre-curfew developments. This variable is equal to one from seven days before the curfew actually starts until the “Effective curfew” ends. Before and after it is equal to zero. We label this variable “Incidence lead”. In formal terms:

2 $$\begin{aligned} \begin{aligned} \text {Incidence lead}_{i,t}= {\left\{ \begin{array}{ll} 1, & \text { if }t\in [\text {Actual curfew start date}_{i} - \text {7 days};\\ & \text {Effective curfew end date}_{i}]\\ 0, & \text {otherwise} \end{array}\right. } \end{aligned} \end{aligned}$$

Loosely speaking, the inclusion of the variable “Incidence lead” tests whether it is plausible to assume that the “common trend assumption” holds.

We further add a dummy which is equal to one for the post-curfew period of the treated counties, formally:

3 $$\begin{aligned} \begin{aligned} \text {After effective curfew}_{i,t}= {\left\{ \begin{array}{ll} 1, & \text { if }t\in [\text {Effective curfew end date}_{i} +\text {1 day};\\ & \text {End of observation period}_{i}]\\ 0, & \text {otherwise} \end{array}\right. } \end{aligned} \end{aligned}$$

Thus, we are able to control whether the growth of incidences differ in the long run.

**Figure 2:**
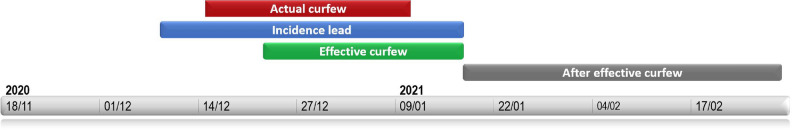
Exemplary illustration of the timeline.

The three variables plus the actual curfew are illustrated in Fig. 2.

The empirical model we estimate can be written as:

4 $$\begin{aligned} \begin{aligned} \frac{I_{i,t}-I_{i,t-1}}{I_{i,t-1}}&=\beta _1 \times \text {Effective curfew }_{i,t} \\&\quad +\beta _2 \times \text {Incidence lead}_{i,t} \\&\quad +\beta _3 \times \text {After effective curfew}_{i,t} \\&\quad +\phi _i+ \phi _i \times \text {Time trend}_t \\&\quad +\gamma _t+\varepsilon _{i,t}, \end{aligned} \end{aligned}$$

where *I* denotes the incidence in county *i* at day *t*. $\beta _1$ is the coefficient of interest – the effect of the night curfew on the growth of incidences *I*. We further include fixed effects for each day in our sample $\gamma _t$ in order to control for general developments of the pandemic spread and for each county $\phi _i$ to control for time-invariant differences across counties that may effect the pandemic such as population density or demographic differences. Additionally, we include interactions of county fixed effects with a linear time trend in order to allow for different general developments over time across counties. Thereby, we accommodate potential trend differentials in our model. This allows us a valid identification of treatment effects even for heterogeneous infection dynamics across regions in the pre-treatment period^10,13,14^. $\varepsilon _{i,t}$ denotes the usual error term. In our empirical analysis we drop the county Waldeck-Frankenberg for two reasons. First, there were two curfews with the second curfew lasting only three days and started only four days after the first one. Second, there were substantial reporting problems during Christmas holidays as incidences shoot up by 209 from December 26 to 27 which is a 387% higher jump than in the county with the second highest jump. In a robustness check we also exclude Christmas holidays and New Year’s day from our data set because there were reporting problems. However, our results remain also fully robust as shown in the “Appendix” (Supplementary Information).

## Results

**Figure 3:**
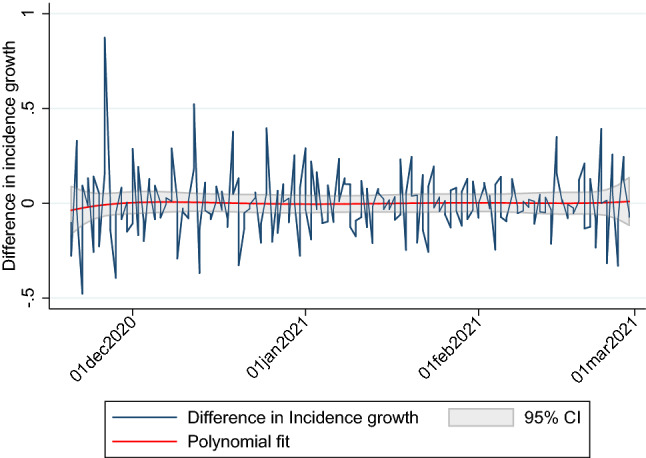
Differences in incidence growth between counties that implemented night curfews and those that did not.

Before we present the results from the econometric analysis we illustrate the patterns descriptively. Statistically, incidence growth did not differ on average between counties that implemented a curfew and those that did not. It was $-0.24$% on average in counties that implemented night curfews and $-0.23$% in counties that did not, with standard deviations of 10.7% and 11.6%, respectively, during our observation period. We plot the difference in incidence growth between counties that have implemented a night curfew during our observation period and those that did not in Fig. 3.

Additionally, we add a polynomial fit and the corresponding 95% confidence interval. As the confidence interval always covers the 0, the difference is not significantly different from zero.

The results from the regression models from Eq. (4) are shown in Table 3. In Column (1) we assume a delay of seven days between the actual start of the curfew until it gets effective. In Column (2) we assume a delay of fourteen days and twenty-one days in Column (3).

All models suggest that there is no evidence for differences in the dynamics of the virus' spread before the night curfews are implemented as indicated by the insignificant coefficients of “Incidence lead”. In other words we can assume common trends for growth rates of incidences in counties with and counties without night curfews. This is important as it enables a causal assessment whether night curfews did affect incidence growth.

The key variable of interest in this paper is the variable “Effective curfew”. Even though the coefficient of this variable is negative, it is never significant at conventional levels for the different specifications we analyse. Given the discussion of how meaningful a concept statistical significance is (for a recent survey see^15^), we further investigate whether there might nevertheless be a small, but meaningful effect of night curfews by computing minimal detectable effects in the next section.

Note that the coefficient of the variable “After effective curfew” is also never significant. Thus, there do not seem to exist differences in the growth of incidences in the long run. Night curfews do not seem to have had a lasting effect after the curfew had ended.

**Table 3:** Effects of night-time curfews on incidences in Hesse.

	7 Days delay	14 Days delay	21 Days delay
	$\frac{I_t-I_{t-1}}{I_{t-1}}$	$\frac{I_t-I_{t-1}}{I_{t-1}}$	$\frac{I_t-I_{t-1}}{I_{t-1}}$
Effective curfew	$-0.007$	$-0.010$	0.008
	(0.010)	(0.009)	(0.012)
Incidence lead	0.019	0.015	0.018
	(0.014)	(0.015)	(0.012)
After effective curfew	0.031	0.017	0.021
	(0.023)	(0.029)	(0.025)
Day FE	Yes	Yes	Yes
County FE	Yes	Yes	Yes
County $\times$ daily time trend FE	Yes	Yes	Yes
R$^2$	0.13	0.13	0.13
Obs.	2550	2550	2550

Cluster-robust standard errors (clustered on county level) are presented in parentheses. Statistics are significant for $$^{***}p<1\%$$, $$^{**}p<5\%$$, $$^{*}p<10\%$$.

### Minimal detectable effects

**Figure 4:**
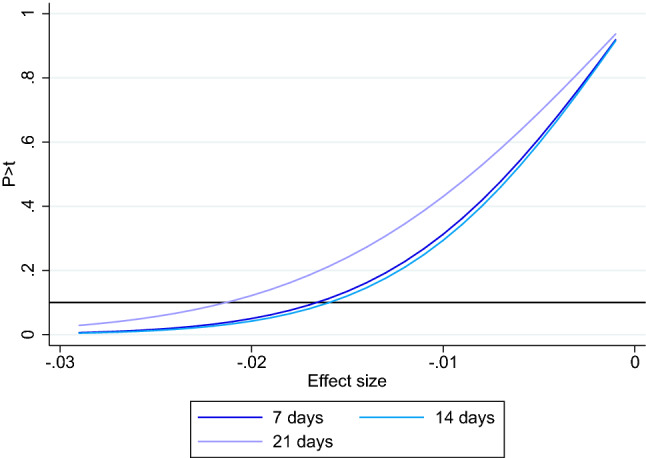
Minimal detectable effects. *Notes*: The solid horizontal line represents the line $10\%$ significance level.

The effects we found for “Effective curfew” are small and non-significant at conventional levels. However, our estimates may suffer from Type II error due to potentially insufficient statistical power. We therefore compute the *minimal detectable effects* (MDE) now. The MDE analysis suggests that with our data we can detect declines in incidence growth if they are smaller than $-1.5$% at the 10% significance level for the model version with 7 days delay and $-1.6$% and $-2.1$%, respetively, for delays of 14 and 21 days. The whole ranges of detectable parameters are shown in Fig. 4. As the variable incidence growth has a standard variation of 10.7 these values are still very small. This supports the finding that night curfews had at best a limited effect on incidence growth.

### Heterogeneous effects

We next analyze whether there were heterogeneous effects of the night curfew. We do this by re-estimating the model from Eq. (4) but this time with individually estimated parameters for each day included in the variable “Incidence lead” and individually estimated parameters for each of the first seven days of “Effective curfew” plus a further dummy which is equal to one for all remaining days of the effective curfew and a dummy which is equal to one for all days of the post-curfew period.

In other words,—with delays $X\in \{14,17,21\}$—, the models we estimate can be written as:

5 $$\begin{aligned} \begin{aligned} \frac{I_{i,t}-I_{i,t-1}}{I_{i,t-1}}&= \sum _{T=1}^{X}\beta _{1,T}\times \text {Day} T \text {before effective curfew }_{i,t} \\&\quad +\sum _{T=1}^{7}\beta _{2,T}\times \text {Day} T \text {of effective curfew }_{i,t} \\&\quad +\beta _3\times \text {After effective curfew}_{i,t} \\&\quad +\phi _i+ \phi _i \times \text {Time trend}_t \\&\quad +\gamma _t+\varepsilon _{i,t}, \end{aligned} \end{aligned}$$

**Figure 5:**
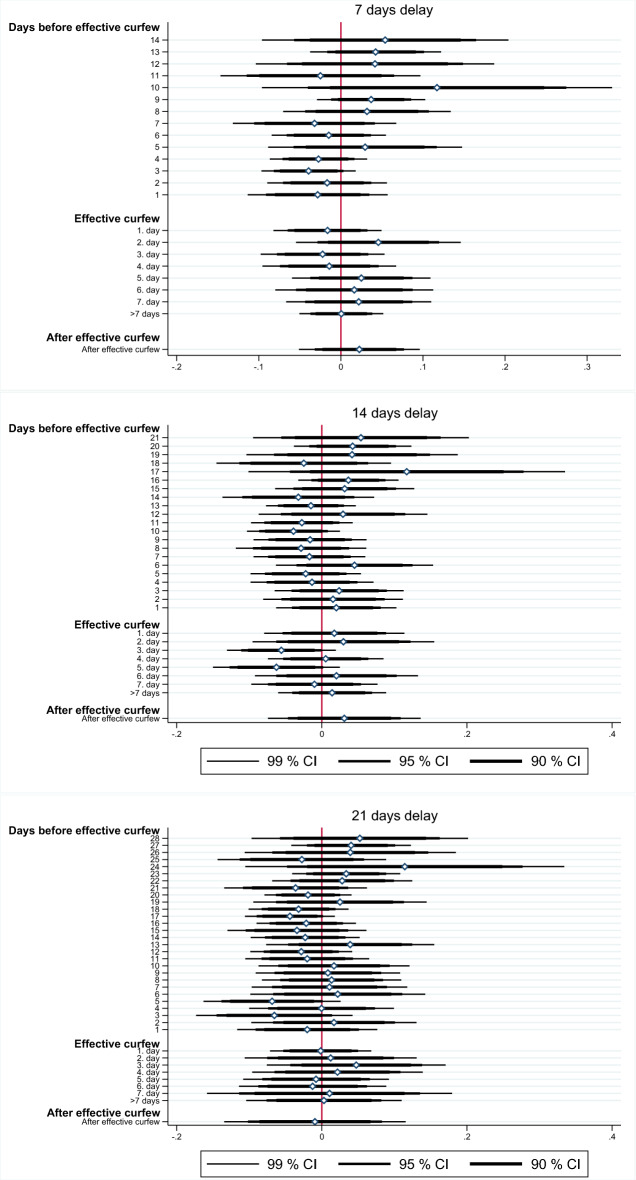
Coefficients and confidence intervals of heterogeneity of effects analysis.

The estimated coefficients and the corresponding confidence intervals of these estimations are presented in the three panels in Fig. 5. Again, the observed patterns do not point towards different trends in the development of incidence growth before the curfew got effective which makes it plausible to assume that the common trend assumption holds. Also, again we do not find any evidence that the night curfews helped to mitigate the spread of the pandemic as all curfew coefficients are statistically insignificant.

## Discussion and conclusion

We estimate the impact of local night curfews in Hesse, Germany, on the growth rates of incidences of COVID-19 cases during the COVID-19 pandemic in this state. While our data set is limited to the federal state of Hesse, the analysis takes advantage of regional and time variation in the implementation of night curfews. Thus, we are able to overcome potential statistical problems that are related to estimations of benefits of NPIs. Our results suggest that night curfews have at best a limited effect in the fight against virus transmission when various other NPIs are already imposed. The general message is that you cannot take for granted that a night-time curfew has the effects derived in ‘international’ studies (e.g.^2^), which estimate the effects of dozens of NPIs in samples consisting of multitude of countries with very different regions. At the same time, there is no indication that the night curfews from 9 pm to 5 am worsen the epidemic. They do not seem to increase incidences. The latter result seems to be important in connection with recent research based on experimental data^16^ and mobility data^17^, respectively, which show that night curfews might fuel disease dynamics.

An obvious question regarding our findings is whether they are the consequence of a weak effect of a night curfew per se or due to an imperfect implementation. Here one has to note the specific nature of a night curfew. It is very easy to understand, to administer, to implement and, in a superficial view, also to monitor. For an example how the curfew was enforced see^18^. On the other hand, it seems next to impossible, at least in a democracy to prevent that especially young people circumvent the night curfew by simply staying with friends overnight. Violations seem unavoidable.

Of course, usual caveats apply. The results may change with another data set. For instance, night curfews could have different effects for other regions. The same is true for the observation period: Our data cover the Christmas season, where a curfew might have fewer additional effects as people tend to stay home anyway. At the same time, it covers New Year’s Eve where the opposite holds. Note, however, that these problems cannot easily be solved by gathering further data and expanding the data set to all of Germany over the full time span of the pandemic. First, measures and policies have been taken and implemented on the level of the German federal states (“Länder”). There was quite some difference with respect to these policies across the states. In the public debate there was a lot of discussion about possibly adverse effects of such a ‘patchwork’ of regulations. Even though such a variance might in principle be a good thing for the researcher, in the case at hand there are too many different determinants which seem to matter. Hesse, for instance, does not border on any foreign country. In the second wave, e.g., incidences in those counties of Bavaria, Thuringia and Saxonia, which are located next to the border to the Czech Republic had particularly high incidences due to the high incidences in the Czech Republic, given a large number of commuters. At the same time, an extension of the observation period to the third wave seems not warranted, even though night-time curfews had also been implemented then. In the third wave vaccinations already had been available but the roll-out also differed across federal states in particular due to administrative reasons^19^. Different to anecdotal evidence from other federal states, where charismatic mayors seem to have had an effect on incidences, no such differences in local governance quality were reported. Hesse in general appears to be rather homogenous in terms of compliance with NPIs.

Finally, it should be emphasized that other minor NPIs such as alcohol ban in certain major urban areas or limitations of indoor individual sports (e.g. tennis) have been introduced simultaneously with night curfews. Thus, theoretically it is possible that some of these measures increase while others decrease incidence growth and sum up to null results. However, while this possibility cannot be excluded it may be a rather unrealistic explanation of our findings.

## Supplementary Information

Supplementary Information.

## Data Availability

All data generated or analysed during this study are included in this published article and are available publicly at the sources referred to, respectively (see references^8,9^).
