# Supplementary material for: Measuring the effect of COVID-19-related night curfews in a bundled intervention within Germany
Source: Sci Rep. 2022 Nov 17;12:19732. doi: 10.1038/s41598-022-24086-9 (PMC9669542; doi:10.1038/s41598-022-24086-9)
Supplement: Supplementary file 1 — Supplementary Information. [file 41598_2022_24086_MOESM1_ESM.pdf]

APPENDIX

Measuring the effect of COVID-19-related night curfews in a bundled intervention within Germany

Samuel de Haas and Georg Götz

Justus-Liebig-University Giessen

Sven Heim

Mines ParisTech

TABLE A1: Effects of night-time curfews on incidences in Hesse – Excluding Christmas holidays and New Year's eve

	7 days delay $\frac{I_t - I_{t-1}}{I_{t-1}}$	14 days delay $\frac{I_t - I_{t-1}}{I_{t-1}}$	21 days delay $\frac{I_t - I_{t-1}}{I_{t-1}}$
Effective curfew	-0.017	-0.016	-0.001
	(0.013)	(0.012)	(0.011)
Incidence lead	0.007	0.003	0.014
	(0.015)	(0.013)	(0.011)
After effective curfew	0.002	-0.009	0.005
	(0.025)	(0.021)	(0.018)
Day FE	Yes	Yes	Yes
County FE	Yes	Yes	Yes
County × Daily Time Trend FE	Yes	Yes	Yes
R^2	0.12	0.12	0.12
Obs.	2,522	2,522	2,522

Notes: Cluster-robust standard errors (clustered on county level) are presented in parentheses. Statistics are significant for $^{***}p < 1\%$, $^{**}p < 5\%$, $^{*}p < 10\%$.
